# WebCARMA: a web application for the functional and taxonomic classification of unassembled metagenomic reads

**DOI:** 10.1186/1471-2105-10-430

**Published:** 2009-12-18

**Authors:** Wolfgang Gerlach, Sebastian Jünemann, Felix Tille, Alexander Goesmann, Jens Stoye

**Affiliations:** 1 Faculty of Technology, Bielefeld University, Bielefeld, Germany; 2 Center for Biotechnology (CeBiTec), Bielefeld University, Bielefeld, Germany; 3 Department of Periodontology, University Hospital Münster, Münster, Germany

## Abstract

**Background:**

Metagenomics is a new field of research on natural microbial communities. High-throughput sequencing techniques like 454 or Solexa-Illumina promise new possibilities as they are able to produce huge amounts of data in much shorter time and with less efforts and costs than the traditional Sanger technique. But the data produced comes in even shorter reads (35-100 basepairs with Illumina, 100-500 basepairs with 454-sequencing). CARMA is a new software pipeline for the characterisation of species composition and the genetic potential of microbial samples using short, unassembled reads.

**Results:**

In this paper, we introduce WebCARMA, a refined version of CARMA available as a web application for the taxonomic and functional classification of unassembled (ultra-)short reads from metagenomic communities. In addition, we have analysed the applicability of ultra-short reads in metagenomics.

**Conclusions:**

We show that unassembled reads as short as 35 bp can be used for the taxonomic classification of a metagenome. The web application is freely available at http://webcarma.cebitec.uni-bielefeld.de.

## Background

Metagenomics is a new field of research on natural microbial communities strongly enhanced by the new high-throughput sequencing (HTS) technologies like Roche's 454-sequencing, ABI's SOLiD or Illumina's Genome Analyzer. In traditional genomics, the sequencing of new microbial genomes was limited to those microbes that can be cultivated in a monoculture, which constitute less than 1 percent of all microbes. To explore the other 99 percent, metagenomics makes it possible to retrieve information about the functional and taxonomic composition of the community they originate from [1].

With decreasing sequencing costs, shotgun metagenomics, i.e. sequencing of random genomic DNA fragments from natural microbial communities, has become feasible [2-4]. Comparing such metagenomic sequences with sequences of known function makes it possible to analyse the biological diversity and the underlying metabolic pathways in microbial communities.

To infer the taxonomic origin of metagenomic reads, two kinds of methods, composition-based and comparison-based, can be distinguished. The composition-based methods extract sequence features, like GC content or *k*-mer frequencies, and compare them with features of reference sequences with known taxonomic classifications [5-7]. In detail, different techniques, like the calculation of correlation coefficients between oligonucleotide patterns [8], self-organizing maps (SOMs) [9], or support vector machines (SVMs) [10] can be used to classify the metagenomic fragments. The comparison-based methods rely on homology information obtained by database searches, e.g. using search tools like BLAST[11,12]. An additional challenge is the fact that the new sequencing techniques produce short (100-500 bp with 454) and ultra-short (35 bp with SOLiD, 35-100 bp with Illumina, 30-35 bp with Helicos [13]) reads. New bioinformatic tools have to be developed that can cope with both, the huge amount of data and the short read lengths. Especially the short read lengths have been considered the main bottleneck for the usage of ultra-short reads in metagenomics [14].

In 2008, we developed CARMA [15], a new software pipeline for the characterisation of species composition and the genetic potential of microbial samples using short reads. In contrast to the traditional 16S-rRNA approach for taxonomical classification [16-18], CARMA uses reads that encode for known proteins. By assigning the taxonomic origins to each read, a profile is constructed which characterises the taxonomic composition of the corresponding community. Krause et al. [15] showed on a synthetic metagenome that even with reads as short as around 100 bp, high accuracy predictions with an average false positive rate of 0.1 to 2.5 percent are possible. The CARMA pipeline has already been successfully applied to 454-sequenced communities including the characterisation of a plasmid sample isolated from a wastewater treatment plant and other communities [19-22].

Here we introduce WebCARMA, a refined version of CARMA available as a web application for the taxonomic and functional classification of unassembled (ultra-)short reads from metagenomic communities. The web application is freely available at http://webcarma.cebitec.uni-bielefeld.de. Furthermore, we examined the applicability of CARMA for simulated ultra-short reads (≥ 35 bp) by comparing the results with earlier ones obtained by short reads (≈230 bp) using samples from a biogas plant [21].

## Implementation

WebCARMA is based on a new CARMA version 2.1 with some improvements over the last published version 1.2 [15]. We first review the basic concepts behind CARMA, before we present our new WebCARMA.

### CARMA

Next-generation sequencing technologies in metagenomics produce millions of DNA reads, from which CARMA detects those that encode for known proteins. These reads are called *environmental gene tags *(EGTs) [23]. In a second step they are assigned to taxa from six taxonomical ranks: superkingdom, phylum, class, order, genus and species. The set of classified EGTs provides a taxonomical profile for the microbial community.

In detail, BLASTX [24,25] is used to search within the set of reads for candidate EGTs that encode for protein sequences contained in the Pfam database [26]. A rather relaxed E-value of 10 and frameshift option -w 15 are used. Each read that has a match to a protein family member is translated according to BLASTX reading frame and frameshift predictions. The final determination of EGTs is done by matching the candidate EGTs against their matching protein families with the corresponding Pfam Hidden Markov Models [27]. For this purpose mmpfam from the HMMER package [28] is used. Only candidate EGTs with an hmmpfam E-value match of 0.01 or lower are accepted as EGTs.

After the EGTs are identified, they are taxonomically classified. Therefore, each EGT is aligned against the multiple alignment of its family with hmmalign (also contained in the HMMER package). From this new alignment, the pairwise sequence distance is computed for all pairs of sequences, based on the fraction of identical amino acids. This produces a pairwise distance matrix which then is used to compute a phylogenetic tree with the neighbour-joining clustering method [29]. The EGT is then classified depending on its position within this tree. If the EGT is localised within a subtree of family members all sharing the same taxon, then the EGT is classified with the same taxon. For example, if the EGT is localised in a subtree with the three members *Bacteria Cyanobacteria Synechococcales Prochlorococcus*, *Bacteria Cyanobacteria Chroococcales Synechococcus *and *Bacteria Cyanobacteria Nostocales Nostoc*, the EGT is classified as *Bacteria Cyanobacteria*. For a more formal definition and further details see [15].

### WebCARMA

We have reimplemented large parts of CARMA, including a faster construction of the phylogenetic trees by caching the pairwise distances between Pfam family members. The CARMA results now include for each EGT the corresponding hmmpfam E-values and a list of GO-Ids (Gene Ontology Identifiers) [30] associated with the corresponding Pfam family. The Gene Ontology provides a controlled vocabulary for gene products, distinguishing between their associated biological processes, cellular components and molecular functions, and can therefore be used to create a functional profile of the metagenome. A major modification of CARMA is the usage of the NCBI taxonomy database [31,32] instead of the Pfam nomenclature. The NCBI taxonomy database currently indexes over 200,000 species [33], which are classified in a hierarchical tree structure. Each taxon from the taxonomy is represented as a node in the tree with a unique identifier (tax_id) and its taxonomic rank ranging from superkingdom to subspecies. For compatibility with other applications and databases, the output files of CARMA contain, along with the prettyprint taxa classifications, the corresponding tax_id.

The WebCARMA website is built upon an Apache Web Server using Perl and CGI. The CARMA pipeline is executed on the compute cluster of the Bielefeld University Bioinformatics Resource Facility at the Center for Biotechnology (CeBiTec) using Sun Grid Engine http://gridengine.sunsource.net/. In order to use WebCARMA and to upload metagenomic sequences, the users have to register with their e-mail address. After the uploads are finished, CARMA starts with the search for EGTs and the taxonomical classification. By the time the jobs are completed, the user receives an e-mail with a download address pointing to the results.

We provide several pre-computed data formats that allow the user to explore the results in different ways:

• The translated EGTs, with additional information about the name of the original metagenomic sequence, reading frame, Pfam ID, HMMER E-value and a list of GO-Ids associated with that corresponding Pfam ID.

• A GO-term profile in two variants, as a text data file and visualised as a histogram in PDF format.

• The taxonomical classification results as a text data file.

• A taxonomic profile, once as a text data file and once visualised by histograms for each taxonomic rank in PDF format.

The profile data files as well as the classification results are provided in TSV-format (Tab Separated Values), which makes it easy to import the data into other programs (e.g. spreadsheet) for different visualisation types or any other further processing.

We provide Perl scripts for download that can easily be used as templates for own data processing pipelines. A manual with further explanations can be found on the WebCARMA site.

## Results and Discussion

### The WebCARMA Web Application

**Figure 1:**
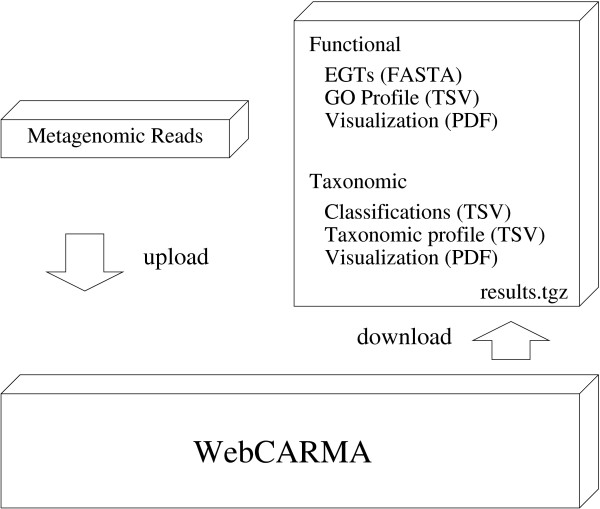
**Overview WebCARMA**. The web application WebCARMA.

A local installation of CARMA [15] requires the Pfam MySQL database and several bioinformatics tools, like BLASTX, the HMMER package, the PHYLIP package [34] and several Perl packages. Most strikingly, it has high computational demands which make the usage of a high-performance grid inevitable. Therefore, we introduce WebCARMA, our new platform-independent web application which makes CARMA easily accessible to the scientific community (see Figure 1).

WebCARMA produces functional (using GO-Terms) and taxonomic profiles (using NCBI taxonomy) of metagenomic sequences, available in text format and as histograms.

**Figure 2:**
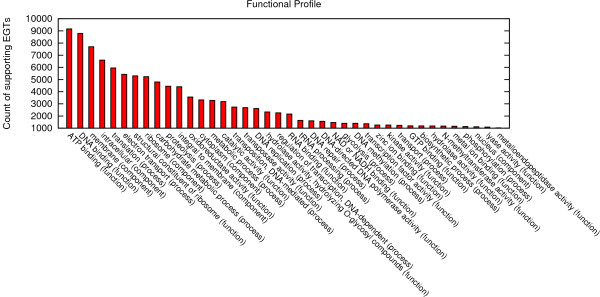
**Functional Profile**. Example of a functional profile: 40 most abundant GO-terms in the metagenome of an agricultural biogas reactor.

An example of a functional profile of a complete metagenomic 454 data set (described in the use case study below) produced with WebCARMA is depicted in Figure 2. Examples of comparative taxonomic profiles are discussed in the use case study.

To classify a metagenomic data set as used in the use case study below (600 000 reads and average 230 bp read length) takes about 1 800 CPU hours (each CPU with 2.5 GHz) on our compute cluster. Because the neighbour-joining algorithm has quadratic runtime, cluster jobs which process bigger Pfam families will take much more time to compute than other. Therefore, using 100 CPUs, it takes for this data set about 40 hours until the last job has finished.

### Comparison of full-length 454 and simulated ultra-short reads

In order to evaluate the applicability of short and ultra-short reads in metagenomics, we used a real-world data set and several realistic simulated data sets. The real-world data set consisted of 600 000 reads with an average read length of 230 bp of a microbial community from an agricultural biogas reactor [35], sequenced with the 454 *Genome Sequencer FLX *system (Roche Applied Science). Instead of using real ultra-short reads, we decided to simulate ultra-short reads by clipping off suffixes of the 454-reads to get the desired read lengths. We generated nine data sets, each consisting of reads of one of the lengths 35 bp, 40 bp, 50 bp, 60 bp, 70 bp, 100 bp, 150 bp, 200 bp, and 250 bp, respectively.

It is known that some sequencing techniques exhibit correlations between read coverage and GC content [36-38]. By using simulated reads instead of real ultra-short reads we can be sure that any differences we see in the classification results between the data sets are only due to the different read lengths. If there is a bias in the 454 data, then we also have the same bias in the simulated data sets and our comparison should not be much affected by this.

First, we analyse the number and lengths of the EGTs obtained for each data set, then we compare the taxonomic classification results for the different read lengths.

As shown in Table 1, the number of reads in each data set decreases with increasing read length. This is because the 454 data set contains reads of different lengths and some of the reads are already too short to serve as a template for all simulated data sets. The relative amount of EGTs that is found in each data set increases with increasing read length.

**Table 1:** Number of reads in each data set.

Length	35 bp	40 bp	50 bp	60 bp	70 bp	100 bp	150 bp	200 bp	250 bp	original
Reads	616 069	616 031	613 943	606 760	598 811	584 168	550 945	492 305	297 852	616 072
EGTs	886	7 836	29 999	48 472	62 112	92 000	119 674	130 544	89 979	172 461
Unique	886	7 827	29 923	48 218	61 687	90 854	116 743	125 624	85 565	164 444
Yield	0.14%	1.27%	4.87%	7.95%	10.30%	15.55%	21.19%	25.52%	28.73%	26.69%

Number of reads and EGT yield for each data set. Some metagenomic reads have matches to more than one Pfam family and therefore are translated into more than one EGT. The column "Unique" denotes the total number of EGTs where EGTs from the same read are counted only once. The column "Yield" denotes the fraction of (unique) EGTs that could be obtained from the corresponding data set.

**Figure 3:**
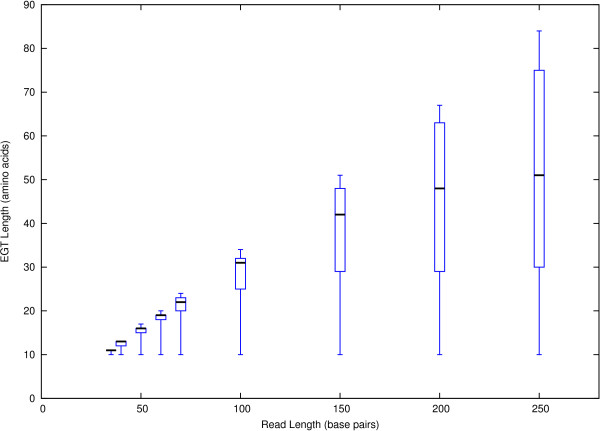
**EGT lengths distribution**. EGT length distribution in each data set as a function of read length. Shown are the minimum, 25% quantile, median, 75% quantile and maximum.

Figure 3 shows the EGT length distribution in each data set as a function of read length. Shown are the minimum, 25% quantile, median, 75% quantile and the maximum. Our results show that the median EGT length does not scale linearly with the read length. The length of Pfam families and domains poses an upper bound on the possible length of local alignments between translated reads and Pfam families. The longer a read is, the higher is the probability that parts of the reads lie outside of the matching gene and can not contribute to the EGT.

In rare cases, it happens that an EGT is one amino acid longer than its read length divided by three. For example, in the set of EGTs produced from the 150 bp-reads data set, the longest EGTs are 51 amino acids long. This can occur when blastx predicts two frameshifts in one read.

For our analysis of the applicability of ultra-short reads with CARMA, we considered seven different taxonomic ranks: superkingdom, phylum, class, order, family, genus and species. A first relative abundance for each taxon is obtained by dividing the absolute number of EGTs that predict this taxon by the total number of EGTs at the same taxonomic rank. The latter do not include EGTs which predict nothing ("unknown"). We consider taxa with a relative abundance below the threshold 0.015 in all data sets, to be false positives. Therefore they are classified as "other".

After applying the threshold we recompute the relative abundances for each taxon, this time subtracting both, "unknown" and "other" from the total number of EGTs at the same taxonomic rank.

With this, we have normalised the relative abundances for the taxa such that they sum up to 1 and therefore ensured comparability between the data sets.

For scaling reasons, the fractions of "unknown" and "other" EGTs are not shown in the histograms (except "unknown" on superkingdom level). This data is given in Tables 2 and 3.

**Table 2:** Rate of "Unknown" EGTs. Rate of "Unknown" EGTs that could not be classified further from the complete set of EGTs.

Read Length	Superkingdom	Phylum	Class	Order	Family	Genus	Species
35	0.09	0.31	0.38	0.45	0.52	0.53	0.59
40	0.09	0.26	0.37	0.43	0.51	0.52	0.57
50	0.09	0.27	0.38	0.43	0.51	0.52	0.58
60	0.09	0.28	0.39	0.45	0.53	0.54	0.61
70	0.09	0.29	0.4	0.46	0.54	0.56	0.63
100	0.1	0.32	0.43	0.49	0.58	0.6	0.68
150	0.11	0.33	0.44	0.52	0.6	0.62	0.71
200	0.11	0.34	0.45	0.52	0.61	0.63	0.73
250	0.11	0.32	0.44	0.51	0.6	0.63	0.73

**Table 3:** Rate of "Other" EGTs.

Read Length	Superkingdom	Phylum	Class	Order	Family	Genus	Species
35	0.0011	0.0671	0.1651	0.2816	0.4057	0.4554	0.6776
40	0.0011	0.0678	0.1691	0.2901	0.4388	0.5056	0.7340
50	0.0019	0.0667	0.1609	0.2954	0.4591	0.5292	0.7606
60	0.0024	0.0619	0.1552	0.2864	0.4637	0.5302	0.7663
70	0.0023	0.0617	0.1554	0.2954	0.4535	0.5221	0.7505
100	0.0035	0.0655	0.1539	0.2891	0.4456	0.4978	0.7172
150	0.0071	0.0692	0.1565	0.2964	0.4555	0.5006	0.6756
200	0.0100	0.0651	0.1467	0.2938	0.4500	0.4954	0.6658
250	0.0137	0.0542	0.1377	0.2849	0.4317	0.4693	0.6364

"Other" are EGT's with a relative abundance below the threshold 0.015 and are not shown in the histograms. Here we show the rates of "Other" EGTs relative to the total number of classified EGTs for each taxonomic rank and data set.

Even though the taxonomic predictions on lower taxonomic ranks (order, family, genus and species) are known to be imprecise, we included them in our experiment in order to study the effect of using (ultra-)short reads compared to longer ones at all taxonomic ranks.

**Figure 4:**
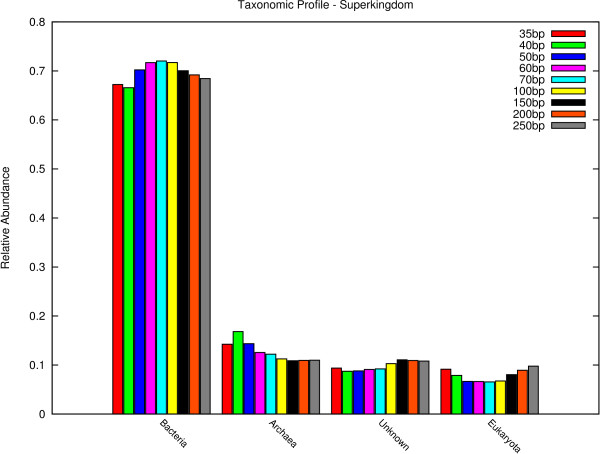
**superkingdom**. Taxonomic results on the level of superkingdom.

**Figure 5:**
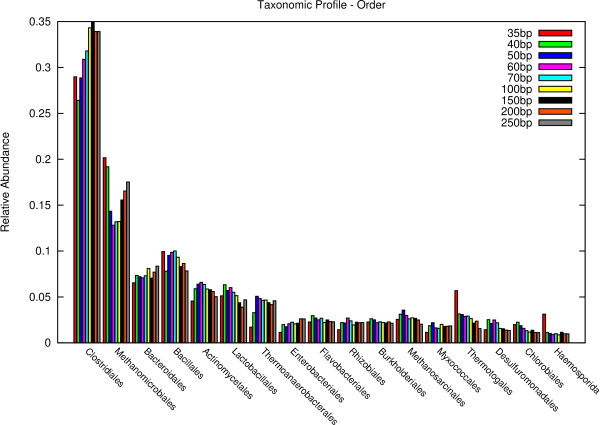
**order**. Taxonomic results on the level of order. Only taxa with an abundance of 0.015 or higher are shown.

**Figure 6:**
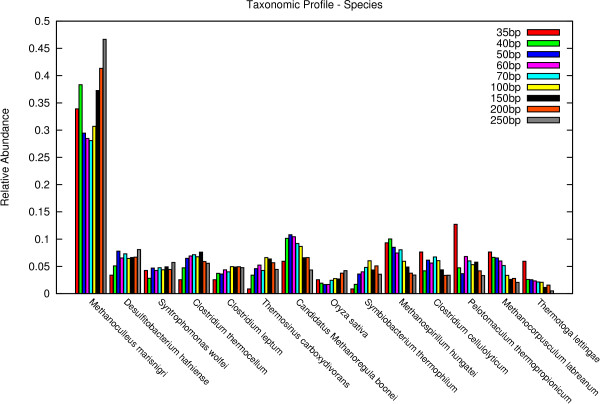
**species**. Taxonomic results on the level of species. Only taxa with an abundance of 0.015 or higher are shown.

Figures 4, 5 and 6 show the results for superkingdom, order and species. The complete set of figures for the evaluation at all taxonomic ranks can be found in Additional Files 1, 2, 3, 4, 5, 6 and 7.

The results show that CARMA predicts for all data sets and all taxonomic ranks the same taxa. For higher taxonomic ranks, even the relative abundance levels are similar between the different data sets. Deviations of 35 bp-reads for example can be seen on the level of order, where significantly more of *Thermotogales *and *Haemosporida*, and less of *Thermoanaerobacterales *are predicted. The 40 bp data set does not show these differences. Even more deviations can be found on lower taxonomic ranks, for example species.

Furthermore, as expected, the rate of EGTs which are not classified increases for lower taxonomic ranks for all data sets (Table 2). Interestingly, the rate of unclassified EGTs is smaller for shorter reads than for longer reads. This might be due to the circumstance that shorter EGTs need to have more sequence similarity to the Pfam families than longer EGTs, in order to achieve the same E-value threshold.

## Conclusions

CARMA is a software pipeline for the characterisation of species composition and the genetic potential of microbial samples using short reads that encode for known proteins. The species composition can be described by classifying the reads into taxonomic groups of organisms they most likely stem from. CARMA has been successfully evaluated on a synthetic metagenome [15] and has already been used for the analysis of several microbial communities [19,20].

Here we have presented our new web application WebCARMA, which makes metagenomic analyses with CARMA easily accessible. WebCARMA provides functional and taxonomic classifications in common data formats as well as basic visualisations of the profiles.

Previous metagenomic analysis relied on reads of length 100 bp or longer. We have shown that ultra-short reads as short as 35 bp can be used for the taxonomic classification of a metagenome. The biogas data set we have used in the analysis is a low complexity data set with only a few prevalent species. Therefore, our results do not necessarily apply to data sets of higher complexity. Still, we think we have shown that ultra-short reads can indeed, in principle, be used for reliable taxonomic classification of a microbial community if the coverage is high enough. We have found most differences between the different data sets in the taxa of higher order, e.g. at the species level, and in general for species with very low abundance.

Metagenomics with CARMA still leaves some room for improvements. Proper statistics to assess the significance of functional and taxonomic predictions based on short reads (Kowalczyk et al.: Significance Tests for Short Read Concentrations, unpublished manuscript) are still missing. The abundance levels of the classification results have to be read with care. Species with larger genomes or more genes than other species will be overrepresented in the taxonomic profiles because more EGTs can be found. Therefore, more accurate results might be achieved by weighting EGTs using additional information like the genome size of the closest known relative.

## Availability and requirements

• **Project name: **WebCARMA

• **Project home page: **http://webcarma.cebitec.uni-bielefeld.de

• **Operating system(s): **Platform independent (web-service); Unix/Linux (stand-alone program)

• **Programming language: **Perl

• **Other requirements: **none

• **License: **GNU GPL

• **Any restrictions to use by non-academics: **none

• **Upload restriction: **Maximum 100 MB of FASTA per month

## Authors' contributions

The new version of CARMA was implemented by WG. SJ and FT contributed to the WebCARMA web application. AG and JS supervised the development of the initial system design and gave important suggestions to the manuscript. All authors have read and approved the manuscript.

## Additional Files

This is the complete set of figures for the evaluation from the use case study. Only those taxa are visualized, where in at least one of both data sets the predicted relative frequency was 0.015 or higher. Taxa that are not visualised contribute to "Other".

## Supplementary Material

Additional file 1 **Taxonomic results on the level of superkingdom.** Only taxa with an abundance of 0.015 or higher are shown. Click here for file

Additional file 2 **Taxonomic results on the level of phylum.** Only taxa with an abundance of 0.015 or higher are shown. Click here for file

Additional file 3 **Taxonomic results on the level of class. **Only taxa with an abundance of 0.015 or higher are shown. Click here for file

Additional file 4 **Taxonomic results on the level of order.** Only taxa with an abundance of 0.015 or higher are shown. Click here for file

Additional file 5 **Taxonomic results on the level of family.** Only taxa with an abundance of 0.015 or higher are shown. Click here for file

Additional file 6 **Taxonomic results on the level of genus.** Only taxa with an abundance of 0.015 or higher are shown. Click here for file

Additional file 7 **Taxonomic results on the level of species.** Only taxa with an abundance of 0.015 or higher are shown. Click here for file
