# Supplementary figures and images for: WebCARMA: a web application for the functional and taxonomic classification of unassembled metagenomic reads
Source: BMC Bioinformatics. 2009 Dec 18;10:430. doi: 10.1186/1471-2105-10-430 (PMC2801688; doi:10.1186/1471-2105-10-430)

Taxonomic Profile - Superkingdom

Relative Abundance

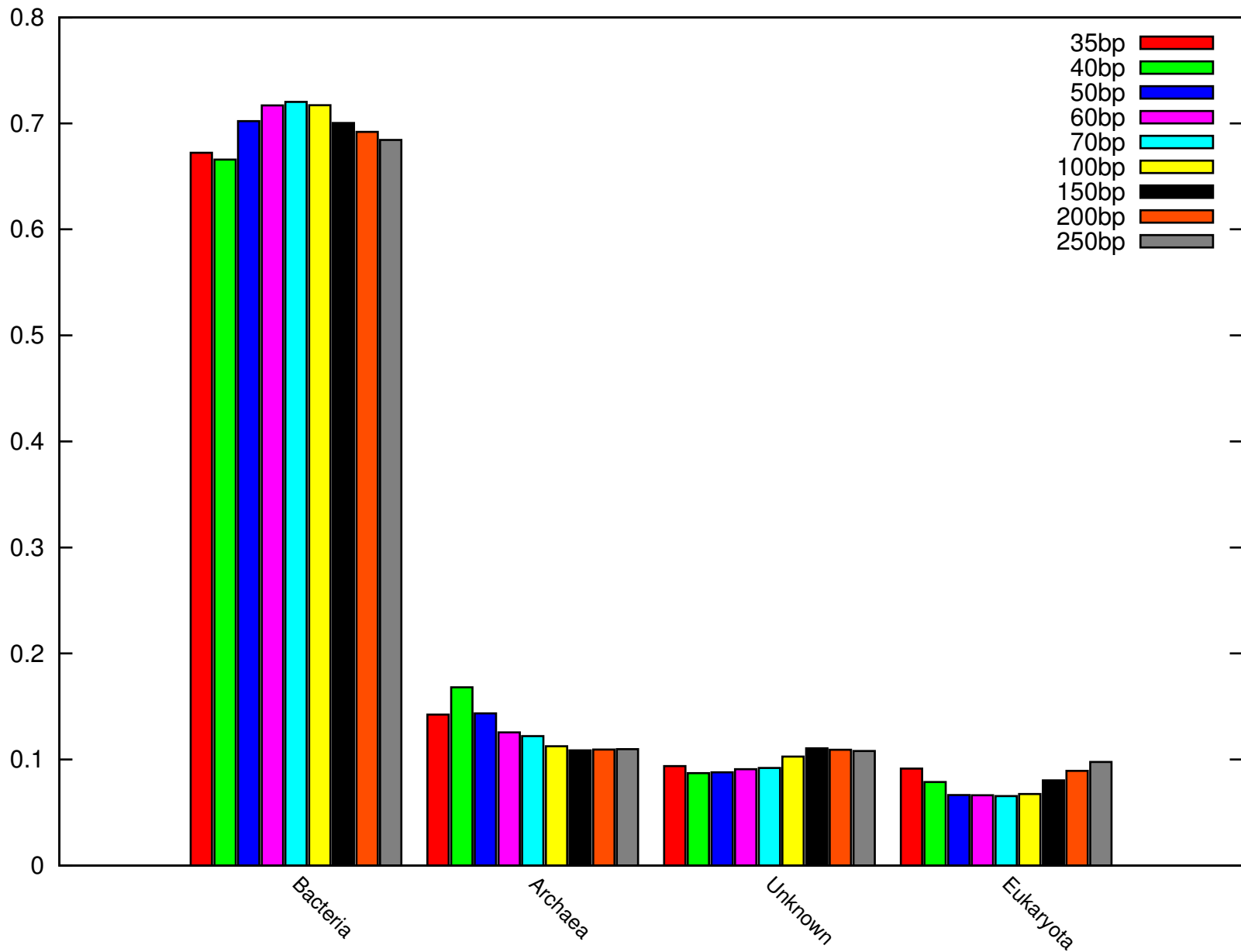

Supplement: Additional file 1 — Taxonomic results on the level of superkingdom. Only taxa with an abundance of 0.015 or higher are shown. [file 1471-2105-10-430-S1.PDF]

Taxonomic Profile - Phylum

Relative Abundance

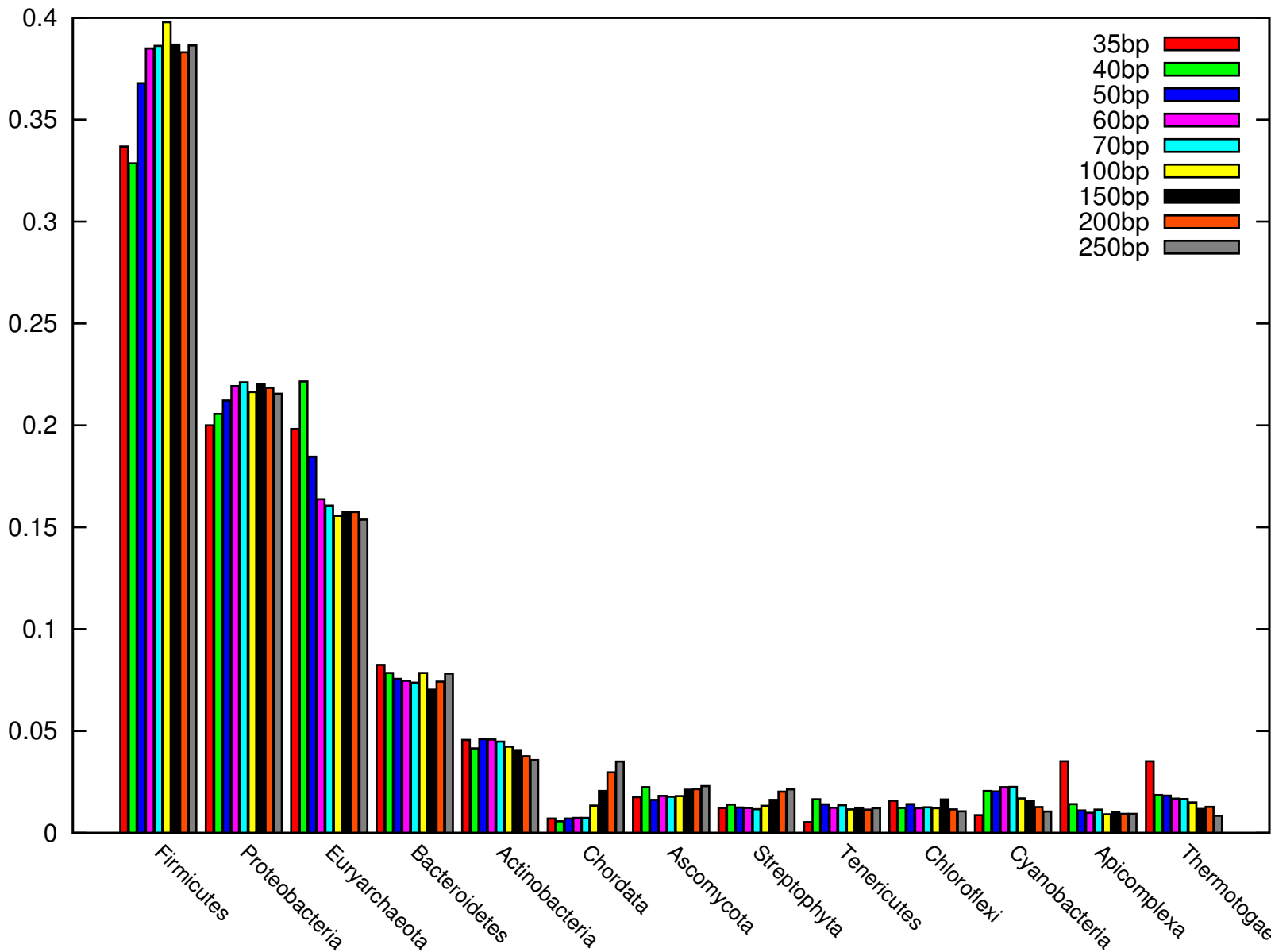

Supplement: Additional file 2 — Taxonomic results on the level of phylum. Only taxa with an abundance of 0.015 or higher are shown. [file 1471-2105-10-430-S2.PDF]

Taxonomic Profile - Class

Relative Abundance

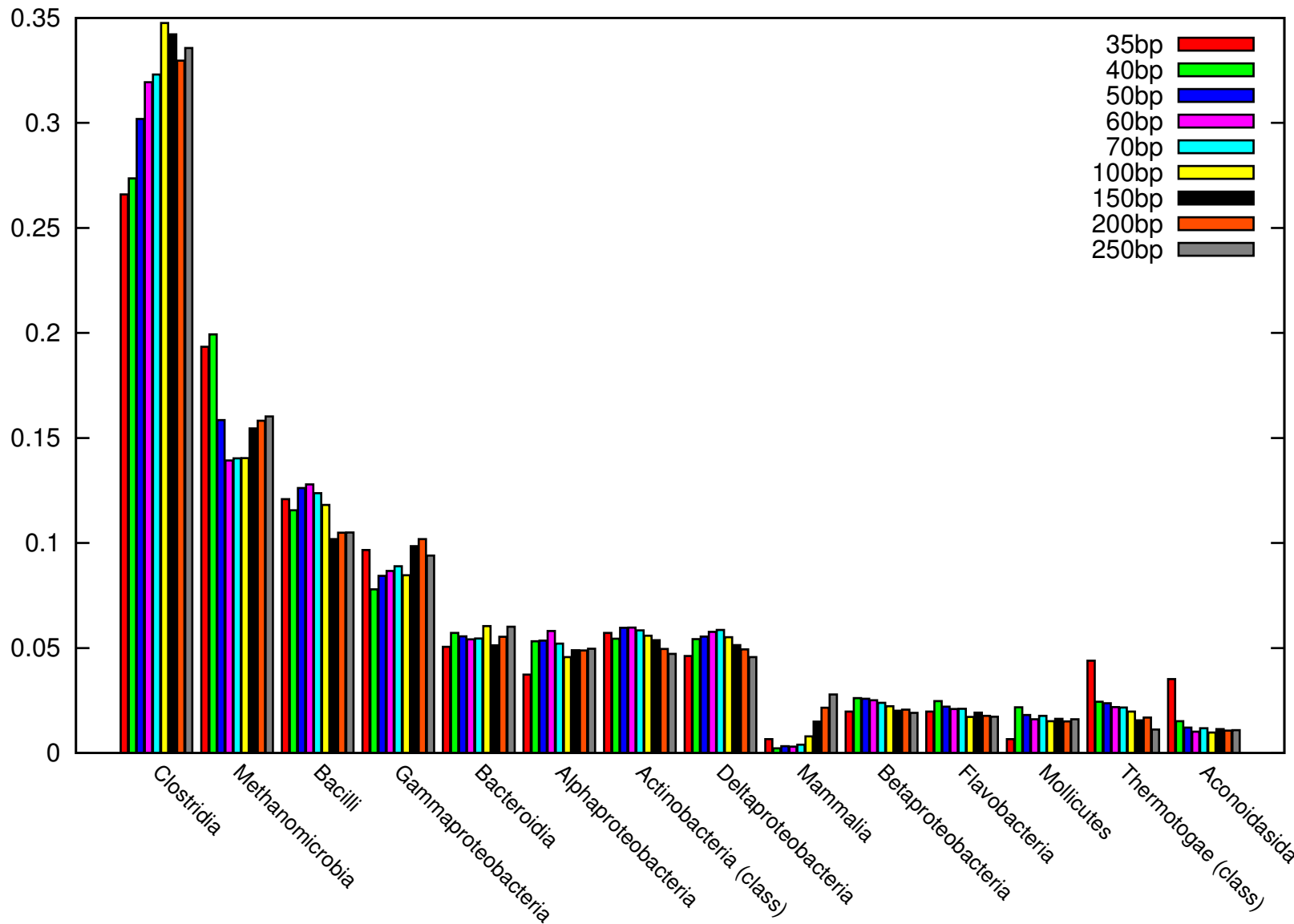

Supplement: Additional file 3 — Taxonomic results on the level of class. Only taxa with an abundance of 0.015 or higher are shown. [file 1471-2105-10-430-S3.PDF]

## Taxonomic Profile - Order

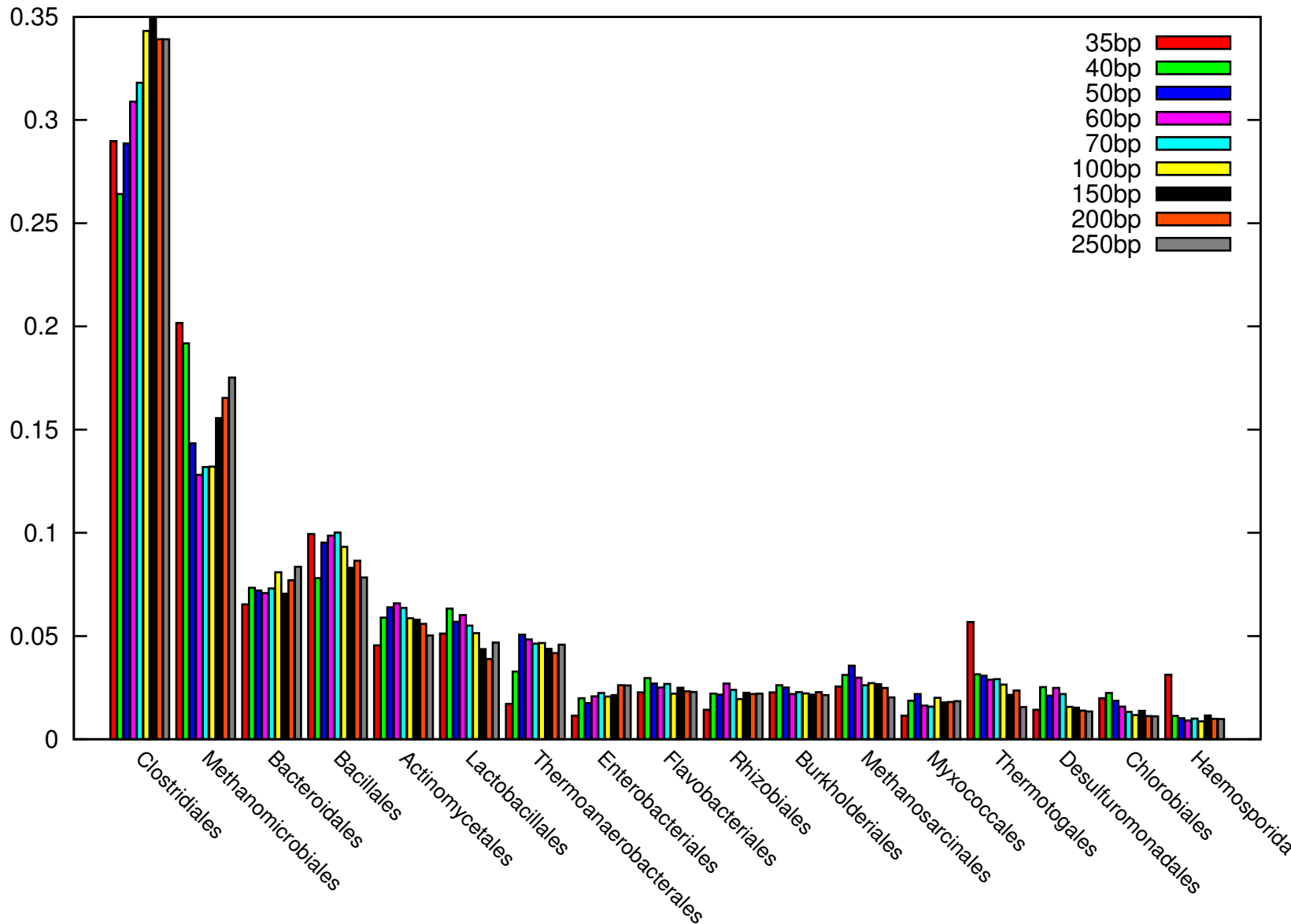

Supplement: Additional file 4 — Taxonomic results on the level of order. Only taxa with an abundance of 0.015 or higher are shown. [file 1471-2105-10-430-S4.PDF]

## Taxonomic Profile - Family

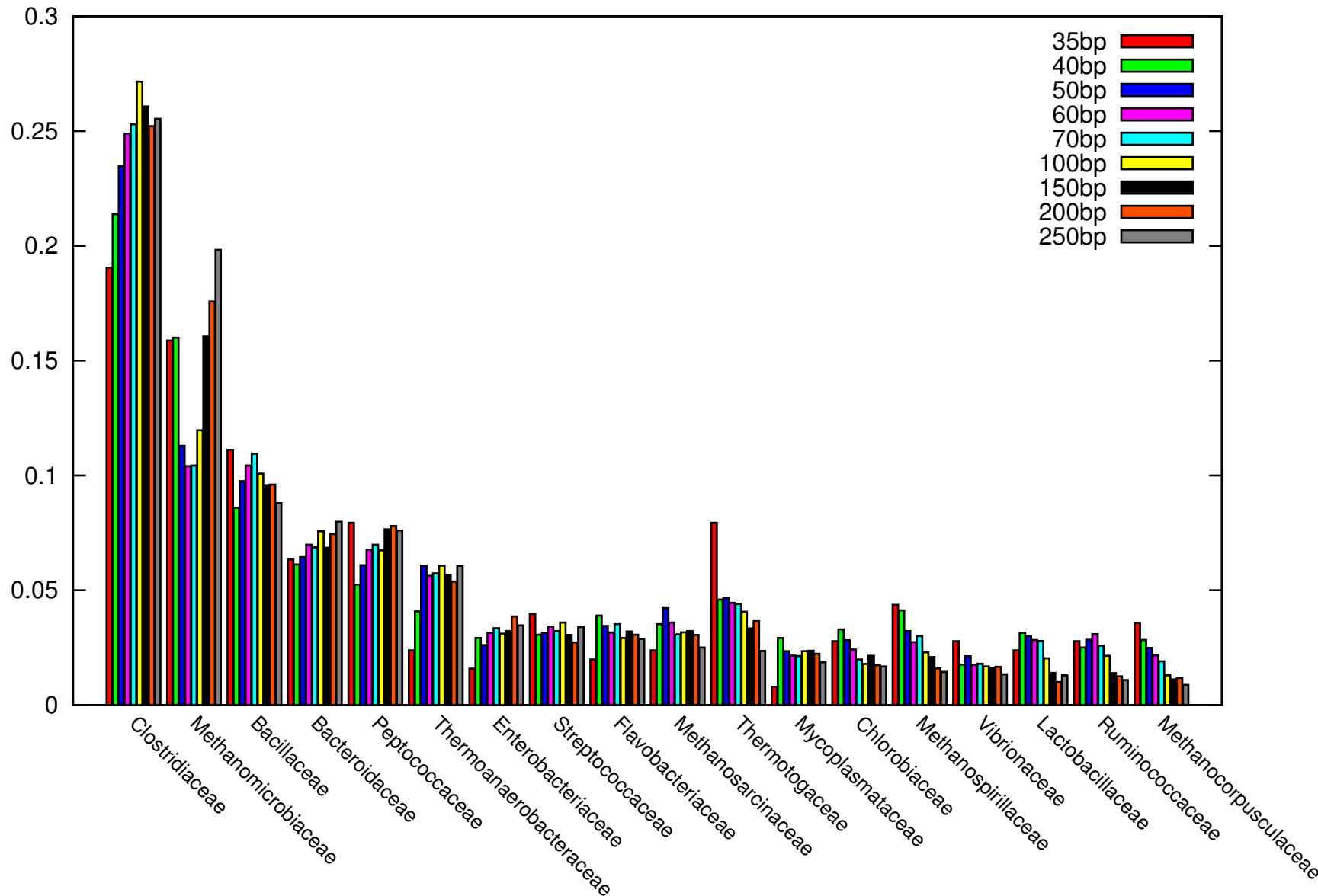

Supplement: Additional file 5 — Taxonomic results on the level of family. Only taxa with an abundance of 0.015 or higher are shown. [file 1471-2105-10-430-S5.PDF]

Taxonomic Profile - Genus

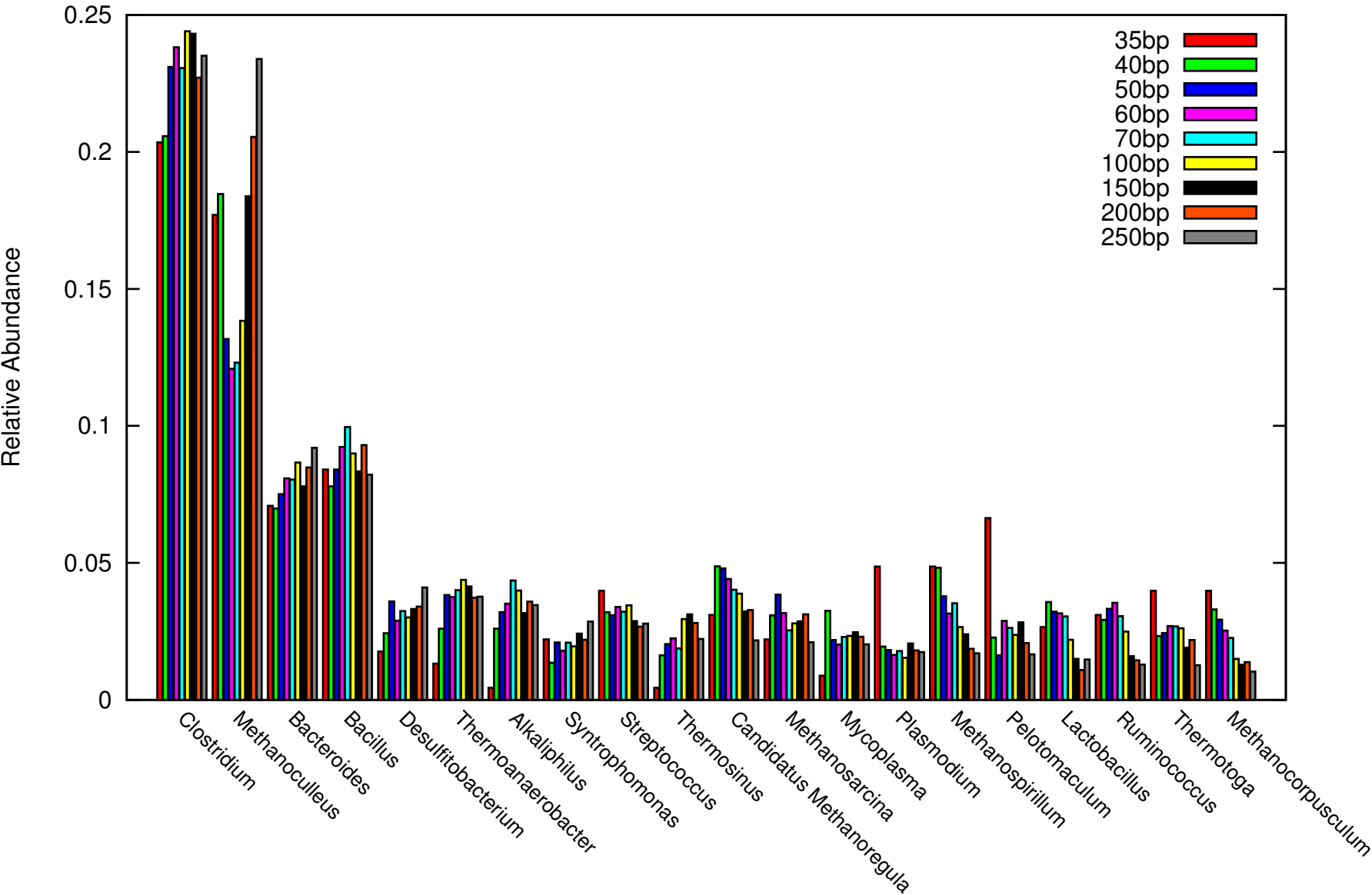

Supplement: Additional file 6 — Taxonomic results on the level of genus. Only taxa with an abundance of 0.015 or higher are shown. [file 1471-2105-10-430-S6.PDF]

## Taxonomic Profile - Species

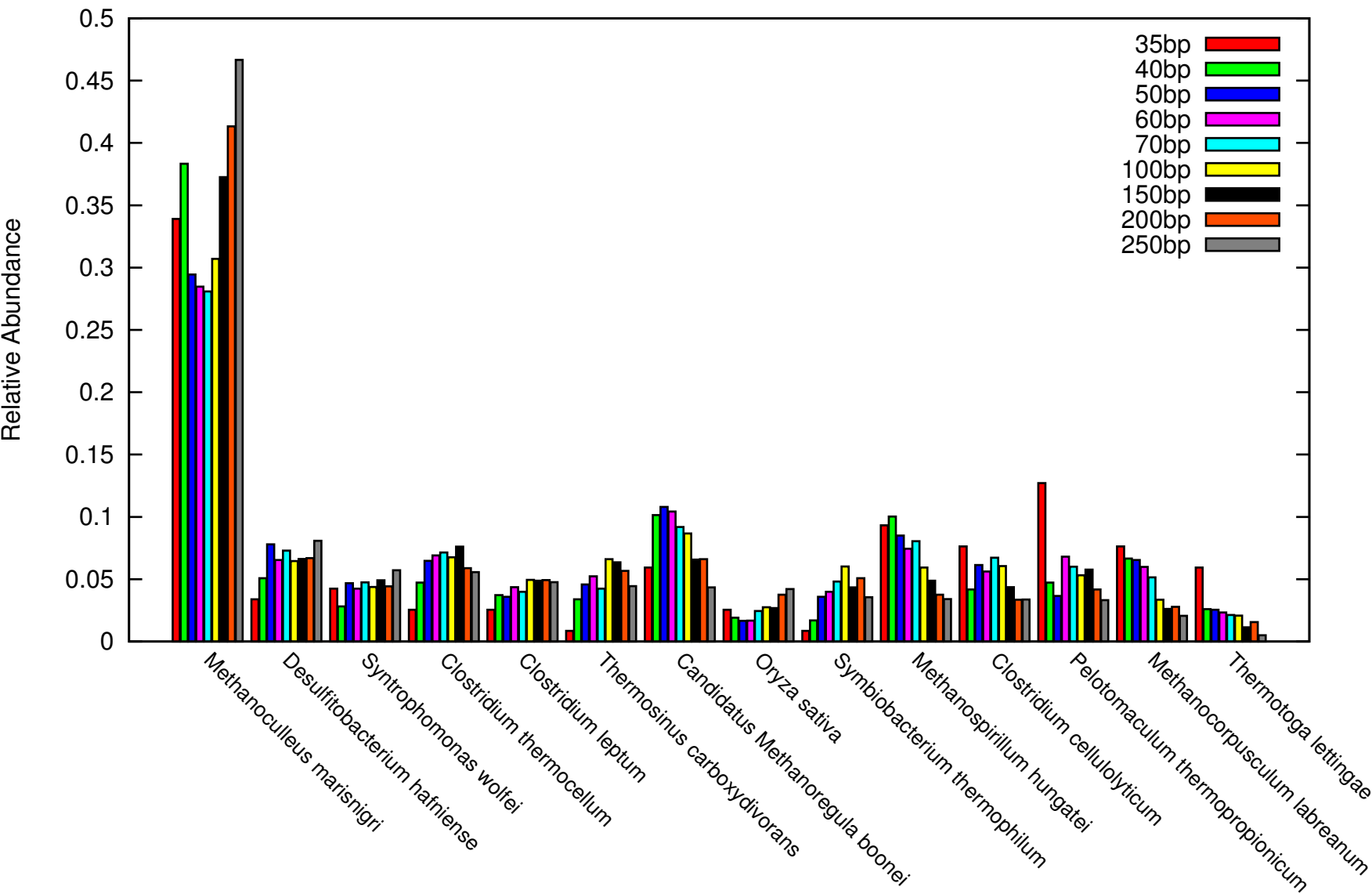

Supplement: Additional file 7 — Taxonomic results on the level of species. Only taxa with an abundance of 0.015 or higher are shown. [file 1471-2105-10-430-S7.PDF]
